# Online Mindfulness with Care Partnerships Experiencing Anxiety and Depression Symptoms after Stroke: Mixed Methods Case Study Research

**DOI:** 10.1177/08980101221135723

**Published:** 2022-11-09

**Authors:** Ben Parkinson, Maggie Lawrence, Evelyn McElhinney, Jo Booth

**Affiliations:** 3525 Glasgow Caledonian University, Glasgow, UK

**Keywords:** stroke, anxiety, depression, mindfulness, online, mixed methods

## Abstract

**Purpose:** To investigate the experience and outcomes for care partnerships (e.g., spouses, caregivers) who have post-stroke anxiety and/or depression symptoms and used an online mindfulness-based intervention (MBI) together. **Design:** Explanatory sequential mixed methods case study research. **Methods:** 5 care partnerships (10 participants) received online MBI, and data was collected in weeks 0, 4, and 8. Data collection involved the Hospital Anxiety Depression Scale, the Mutuality Scale, the Mindful Attention Awareness Scale, and post-intervention interviews. Clinical effectiveness was evaluated using minimal clinically important difference (MCID). **Findings:** Participants improved mindfulness (80%) and mutuality (30%). MCID was achieved for anxiety symptoms (50%) and depression symptoms (20%). IPA found evidence of conflicting and contradictory experiences so dialectical tension was used to articulate the continuum of perspectives and themes produced in the analysis. **Conclusion:** Care partnerships using online MBIs can experience improvements in mindfulness, mutuality, anxiety symptoms, and depression symptoms. The findings are complex but show the potential value of online MBI for some care partnerships living with stroke.

## Introduction

Stroke is the second leading cause of death worldwide and surviving a stroke can result in complications that affect the whole person and result in significant morbidity (Katan & Luft, 2018; Soto et al., 2020). Stroke survivors frequently experience post-stroke affective disorders, which can increase mortality, worsen disability, reduce quality of life, and hinder rehabilitation (Ayerbe, 2015). The prevalence of post-stroke anxiety is estimated at 18.7% (95% CI, 12.5, 24.9) when assessed using clinical interview and 24.2% (95% CI, 21.5, 26.9) when assessed using a rating scale (Knapp et al., 2020). The prevalence of post-stroke depression is estimated to be 31% (95% CI, 28%, 35%) within the first five years post-stroke (Hackett & Pickles, 2014). Recommended treatment for post-stroke anxiety/depression usually involves antidepressants and/or psychosocial interventions (Ayerbe, 2015). Unfortunately, post-stroke anxiety/depression is often undiagnosed, inadequately treated, and clinical outcomes can be poor (Hackett & Pickles, 2014; Ayerbe, 2015; Dar et al., 2017; Ladwig et al., 2018). Mood disorders not only affect the stroke survivor, but are also common among their care partners (e.g., spouses, informal caregivers). Being a care partner can be challenging and often comes with interpersonal problems and concerns about the stroke survivor's wellbeing (Camak, 2015; McCarthy et al., 2020). The prevalence of depression and anxiety among stroke care partners is estimated at 40.2% (95% CI, 30.1, 51.1) and 21.4% (95% CI, 11.6, 35.9) respectively (Loh et al., 2017). A recent critical review explored the relationship between stroke survivor and care partner mental health and found a statistically significant relationship in anxiety and depression, which suggests the mental health of stroke survivors and their care partners is interconnected (Hultman et al., 2019). Holistic nursing practice involves screening stroke survivors and, if appropriate, their caregivers for anxiety/depression symptoms and should aim to support stroke survivors and their care partners together in care partnerships (Bakas et al., 2017).

## Mindfulness-Based Interventions (MBIs)

Mindfulness-based intervention (MBI) describes a variety of different structured mindfulness courses used in clinical and non-clinical settings. All MBIs aim to develop present moment awareness through contemplation and mindfulness meditation. MBIs also incorporate psychological/educational principles, are informed by a model of human distress, and aim to improve self-regulation (Crane et al., 2017). The first MBI course was developed in the 1980s and evolved into mindfulness-based stress reduction (MBSR) (Santorelli et al., 2017). MBSR consists of eight-weekly group sessions and a day retreat. The MBSR course covers formal mindfulness practices (e.g., body scan, yoga, sitting meditation, and walking meditation) and informal practices (e.g., awareness of pleasant/unpleasant events, awareness of breath). MBSR incorporates home practice and participants are encouraged to practice mindfulness regularly between sessions (Santorelli et al., 2017). Another common MBI is mindfulness-based cognitive therapy (MBCT). MBCT shares similarities with MBSR, but also uses elements of cognitive therapy to help people with recurrent depression (Teasdale et al., 2000).

MBIs integrate biological, psychological, social, and spiritual dimensions and promote holistic wellbeing (White, 2014). MBIs are thought to produce therapeutic benefit by helping participants increase their levels of acceptance and mindfulness through regular mindfulness practice (Stein & Witkiewitz, 2020). Research evidence suggests MBIs work effectively in a variety of clinical settings and have a small to medium effect on anxiety and depression when using online technology (Spijkerman et al., 2016). MBI research suggests stroke survivors might gain bio-psychosocial benefit from MBIs, but more robust research is needed before fully understanding the possible benefit of MBIs for people affected by stroke (Lawrence et al., 2013). Recent developments with MBIs include using online technology, involving different clinical populations, and/or delivering MBI in care partnerships (Fish et al., 2016). This study is the first study to examine online MBI with care partnerships experiencing anxiety and depression symptoms following stroke (Parkinson et al., 2019; Parkinson, 2021).

## Aim

To investigate the use of online MBI with stroke survivors and their care partners.

## Methods

**Figure 1.**
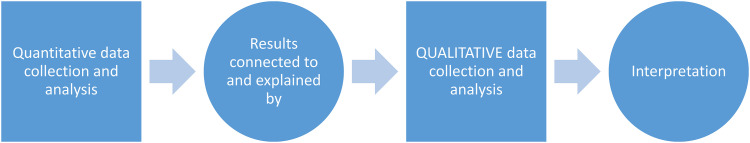
Explanatory sequential design. (Reproduced with permission Creswell and Plano Clark, 2017)

This study aligns with the development phase of the Medical Research Council framework for developing and evaluating complex interventions (Skivington et al., 2021). The study combined an initial quasi-experimental stage followed by Interpretative Phenomenological Analysis (IPA) (Creswell & Plano Clark, 2017; Smith et al., 2009) in an explanatory sequential mixed methods case study design (Figure 1). Mixed methods case study research combines mixed methods research with case study methods and is considered a complex adaptation of mixed methods research (Creswell & Creswell, 2018). Mixed methods case study research allows for data to be analysed at an individual participant level (within case) and at a group level (between cases) (Creswell & Plano Clark, 2017). The quasi-experimental stage used pre/post-test within subject case study design (Flannelly et al., 2018). IPA used an inductive approach to explore the meaning and the ‘lived experience’ of stroke survivors and their care partners using online MBI together (Smith et al., 2009). IPA was used because it focuses on ‘lived experience’ and is flexible enough to capture individual and multiple perspectives (Larkin et al., 2018). The idiographic focus of IPA provided an opportunity for participants to explore the holistic experience of using online MBI as a stroke care partnership (Smith et al., 2009).

## Ethical Considerations

Ethical approval was secured from Glasgow Caledonian University Research Ethics Committee before the study started (HLS/PSWAHS/17/293). All participants provided voluntary informed written consent prior to being enrolled in the study and could withdraw their consent at any time. The study was prospectively registered (NCT03473054).

## Recruitment

Mixed methods case study research using IPA involves identifying a small homogenous group of people with the necessary lived experience to provide an insider perspective on the topic being investigated (Smith et al., 2009). This study purposely recruited five stroke care partnerships (i.e., ten participants) because the number of participants is small enough to maintain an idiographic focus, but large enough to allow for cross-case analysis (Smith et al., 2009). Participants were purposively recruited in 2019 from the community by disseminating adverts on Twitter and through adverts being circulated by voluntary sector organisations (e.g., Stroke Association, Different Strokes, Headway, and Chest Heart Stroke Scotland). Potential participants were invited to make contact and given information about the study. Eligible participants completed consent, were enrolled into the study, and provided with access to an online MBI, Be Mindful (Table 1).

## Intervention

Be Mindful (www.bemindfulonline.com) is a commercially available online MBI, which was provided free of charge to all participants. Be Mindful is delivered online asynchronously over four weeks by two experienced MBI teachers. The content is delivered using ten pre-recorded therapist-led interactive videos, twelve daily practice assignments (with supportive emails), five audio downloads, and online tools for reviewing progress. The instructional videos cover formal mindfulness exercises (e.g., body scan, mindful movement, sitting meditation, and three-minute breathing space) and informal mindfulness exercises (e.g., mindful eating and mindful walking) (Krusche et al., 2012). Be Mindful also has automated data collection for stress, anxiety, and depression. A comprehensive summary of Be Mindful has been published (Krusche et al., 2012; Querstret et al., 2017). A non-randomised single group evaluation of Be Mindful recruited 273 participants using self-referral. The mean age of participants was 47.7 years (SD 11.98, range 20–80) and most participants were female (78%) (Krusche et al., 2013). The evaluation produced a very large effect size for anxiety symptoms post-intervention (d = 1.22) and at follow-up (d = 1.42) and a large effect size for depression symptoms post-intervention (d = 0.95) and at follow-up (d = 1.08) (Krusche et al., 2013). An RCT recruited 118 participants (60 intervention group, 58 control group) from a non-clinical population and compared Be Mindful with a waiting list control (Querstret et al., 2018). The mean age of participants was 40.68 years (SD 10.45, range 21–62) and most participants were female (80.5%). The RCT found a significant reduction in anxiety symptoms (d = −1.09 [−1.47, −0.98]) and depression symptoms (d = −1.06 [−1.44, −0.67]) for participants who completed the intervention (Querstret et al., 2018). Both these studies provide initial evidence for the effectiveness of Be Mindful, but neither study used Be Mindful with care partnerships affected by stroke.

## Data Collection

Data collection occurred during home visits (weeks 0, 4, 8) and when using Be Mindful. Recruitment and retention data were gathered to evaluate feasibility of using online MBI with care partnerships affected by stroke. Engagement with online MBI was evaluated by calculating the number of modules completed by each participant during the study (weeks 0, 4, 8). Adverse events were monitored during the study to highlight possible safety issues.

The primary quantitative measures for mindfulness, mutuality, anxiety, and depression were completed during home visits. The Mindful Attention Awareness Scale (MAAS) includes fifteen questions and is considered a valid and reliable tool for measuring mindfulness, although, it has not been validated for stroke survivors (Brown & Ryan, 2003). The Mutuality Scale (MS) includes fifteen questions and measures love, shared pleasurable activities, shared values, and reciprocity (Archbold et al., 1990). The MS is a valid and reliable tool for measuring mutuality with stroke survivors and care partners (Pucciarelli et al., 2016). The Hospital Anxiety and Depression Scale (HADS) has fourteen questions and is a valid and reliable measure for anxiety and/or depression symptoms (Zigmond & Snaith, 1983). Higher HADS scores indicate greater severity anxiety/depression, and the measure has been validated with stroke survivors (Sagen et al., 2009).

Secondary automated measures for stress, anxiety, and depression were completed by Be Mindful (pre, post, follow-up). The automated measures were the Perceived Stress Scale (PSS) (Cohen et al., 1983), the Generalised Anxiety Disorder (GAD-7) (Spitzer et al., 2006), and the Patient Health Questionnaire (PHQ9) (Kroenke et al., 2001). Automated data collection experienced a large amount of missing data because not all participants completed modules or the automated measures. Due to the high level of automated missing data, a decision was made to focus this report on the primary measures and provide the automated secondary measures as a supplementary file (see Supplementary File).

Post-intervention qualitative in-depth interviews (week 8) were completed with stroke survivors and their care partners together to explore their experience of using online MBI and their views on people affected by stroke using online MBI (Table 2). Interviews were audio recorded and transcribed verbatim before analysis.

**Table 1.** Eligibility Criteria for Stroke Survivors and Care Partners

Inclusion	Exclusion
Stroke survivor or care partner Adults only United Kingdom Access to the internet Can understand English Self-reported anxiety and/or depression symptoms	Difficulty eating Suicidal thoughts Already receiving mental health support Previously attended an MBI Memory or cognitive impairment Hospital in-patient

**Table 2.** Interview Schedule

Topic	Questions
Stroke	Can you tell me how stroke has affected your life?
Intervention	What has the experience of using the course been like?
Mindfulness	What has the experience of learning mindfulness been like?
Online	What has the experience of learning mindfulness online been like?
Partnership	What has the experience of learning mindfulness together been like?
Wellbeing	What changes (if any) have you noticed since learning mindfulness?
Research	What has the experience been like for you both to be involved in this research?
Other	Is there anything else you think is important that I have not asked about?

## Analysis

Descriptive statistics helped understand and explain quantitative data on an individual and cross case basis (Table 3). Recruitment and module completion data was used to evaluate appropriateness and acceptability of online MBI, with higher module completion suggesting greater appropriateness and acceptability for participants. Quantitative results were evaluated using descriptive statistics and direction of effect. Clinical change was evaluated using the HADS and minimal clinically important difference (MCID). The MCID on the HADS for people with cardiovascular disease is >1.7 and was used to indicate possible clinical benefit for participants (Lemay et al., 2019). Item-level missing data was managed using mean imputation methods and unit level missing data was managed using pairwise deletion.

**Table 3.** Results Table

	Modules completed (%)			MAAS			MS			HADS-A			HADS-D		
	Weeks 0, 4, 8			Weeks 0, 4, 8			Weeks 0, 4, 8			Weeks 0, 4, 8			Weeks 0, 4, 8		
Stroke survivor 1	0(0)	4(100)	4(100)	3.64^a^	NR	5.07	3.33	NR	3.33	11	NR	10	6	NR	5
Stroke survivor 2	0(0)	3(75)	4(100)	3.45^a^	4.0^a^	4.87	3.13	3.6	3.93	15	9^b^	8^b^	10	3^b^	3^b^
Stroke survivor 3	0(0)	3(75)	4(100)	4.18^a^	4.27^a^	4	3.6	3.93	3.8	8	9	11	10	5^b^	7^b^
Stroke survivor 4	0(0)	3(75)	4(100)	3.91^a^	4.5	5.13	4	3.8	3.8	9	5^b^	2^b^	3	6	3
Stroke survivor 5	0(0)	1(25)	3(75)	3.53	4.07	4.02	4	4	4	10	11	8^b^	1	3	4
Care partner 1	0(0)	2(50)	2(50)	3.36^a^	NR	NR	3.27	NR	NR	8	NR	NR	5	NR	NR
Care partner 2	0(0)	2(50)	3(75)	4.27^a^	3.63^a^	5.07	3.53	3.53	3.0	4	4	5	0	1	0
Care partner 3	0(0)	3(75)	3(75)	4.45^a^	4.1^a^	5.27	3.67	3.6	3.53	5	7	4	1	0	1
Care partner 4	0(0)	0(0)	0(0)	3.45^a^	4.1	4	3.73	3.47	3.53	5	2^b^	3^b^	2	2	2
Care partner 5	0(0)	0(0)	0(0)	4.27	3.8	4.33	3.8	3.4	3.87	8	4^b^	3^b^	3	3	2

*Note.* HADS-A = Hospital Anxiety and Depression Scale: Anxiety Subscale; HADS-D = Hospital Anxiety and Depression Scale: Depression Subscale; MAAS = Mindful Attention Awareness Scale; MS = Mutuality Scale; NR = not reported; ^a^missing item level data; ^b^ minimal clinically important difference (MCID).

IPA was completed to explain the quantitative findings and to explore the experiences and outcomes for participants (Smith et al., 2009). IPA takes an inductive approach to understand the meaning and experience of people with ‘lived experience’ of the phenomenon under investigation (Smith et al., 2009). IPA is a flexible approach, suitable for nursing research, and can capture multiple perspectives on the same phenomenon (i.e., stroke survivors and their care partners) (McInally & Gray-Brunton, 2021). IPA is influenced by three important philosophical foundations: phenomenology, hermeneutics, and idiography (Smith et al., 2009). IPA in this study involved a series of iterative steps and was completed one transcript at a time to maintain idiographic focus (Table 4).

**Table 4.** Analysis Strategies

Read transcripts and complete explorative commenting Identify emergent themes Search for connections across themes Repeat the process with all transcripts Search for cross-case patterns Use supervisors to review the themes Produce detailed narrative of the analytic process and findings

Developing cross-case themes involved assimilating perspectives into unified concepts but assimilating complex findings can create an oversimplified interpretation of a complex phenomenon (Clark et al., 2012). Dialectics originates from philosophy and provides a useful framework for interpreting complex clinical data or when tension exists between different perspectives (Fagerström, 2006). Dialectics can be used in nursing research as a philosophical position or a method for describing complex processes (Fagerström & Bergbom, 2010). Dialectics were used in this study to describe the complex and often contradictory interpretation of stroke care partnerships using online MBI together (Fagerström & Bergbom, 2010).

## Results

### Participants

**Figure 2.**
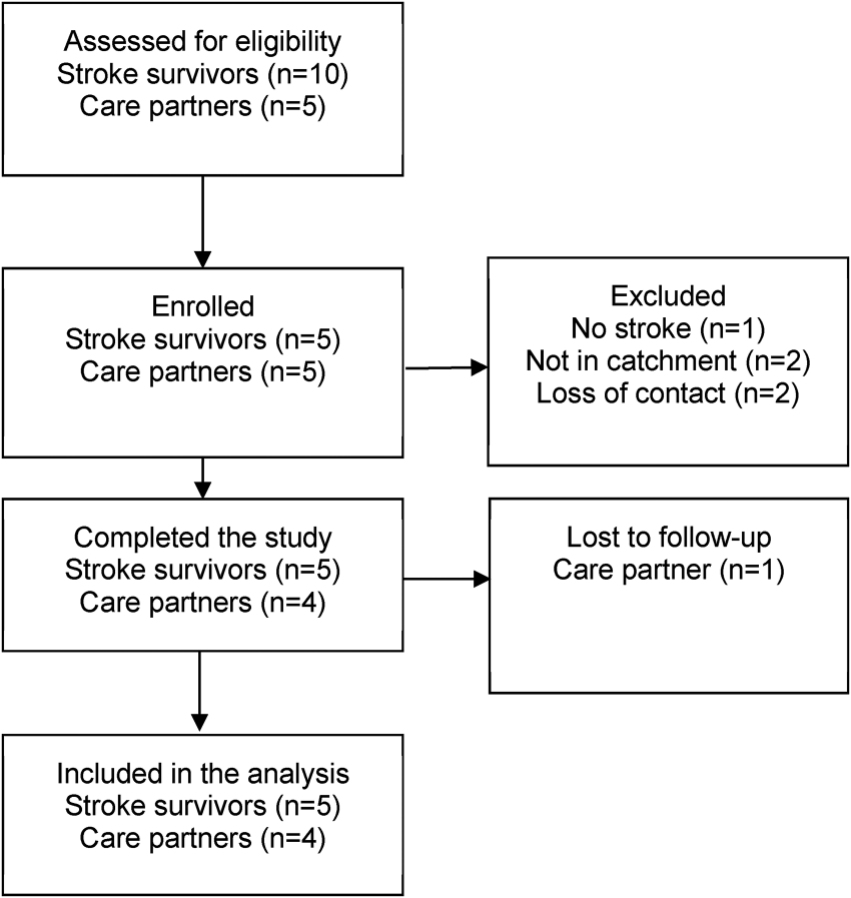
Consort flow diagram (Vohra et al., 2015).

Fifteen people were screened, and ten participants (67%) were enrolled (Figure 2). Participants lived in Scotland (80%) or England (20%) and were aged 40–65 years (mean 53.9 years). The sample was divided between male (50%) and female (50%) and included intimate relationships (i.e., spouses/couples) (80%) and a father-daughter partnerships (20%). Stroke survivors had experienced between one and three strokes within the last nine years. Stroke survivors reported anxiety symptoms (80%), stress symptoms (20%), panic symptoms (40%), and depression symptoms (20%). Stroke survivors reported difficulty with left/right sided weakness (100%), mobility (40%), aphasia (20%), fatigue (20%), memory (20%), and seizures (20%) (Table 5).

**Table 5.** Participant Information

	Age	Sex	Ethnicity	Education	Relationship (years)	Living Together	Employment	Type of stroke (n)	Most recent stroke	Stroke survivor difficulties
Stroke survivor 1 Care partner 1	55 49	Male Female	White Scottish White Scottish	Degree HNC	Couple (15 years)	Yes	Employed Retired	Haemorrhagic (n = 1)	2017	Left-sided weakness Poor mobility Stress and panic
Stroke survivor 2 Care partner 2	57 56	Male Female	White Scottish White Scottish	Degree High school	Couple (21 years)	Yes	Employed Employed	Unsure (n = 2)	2017	Right-sided weakness Poor mobility Anxiety
Stroke survivor 3 Care partner 3	60 40	Male Female	White Scottish White Scottish	HND Degree	Father-daughter	No	Retired Employed	Ischaemic (n = 1)	2017	Right-sided weakness Aphasia Anxiety and depression
Stroke survivor 4 Care partner 4	48 50	Female Male	White Scottish White Scottish	HNC A levels	Couple (8 years)	Yes	Retired Employed	Haemorrhagic (n = 3)	2016	Left-sided weakness Poor memory Fatigue Anxiety
Stroke survivor 5 Care partner 5	59 65	Male Female	White British White British	PgC High school	Couple (18 years)	Yes	Employed Retired	Ischaemic (n = 1)	2010	Left-sided weakness Seizures Anxiety and panic

*Note.* HNC = Higher National Certificate; HND = Higher National Diploma; PgC = Postgraduate Certificate.

### Quantitative Results

Online MBI module completion was higher for stroke survivors than their care partners in every care partnership. Most stroke survivors (80%) completed all modules, whilst none of the care partners completed all modules and some care partners (40%) completed no modules. Online MBI appears more appropriate and acceptable for stroke survivors, compared with their care partners. One serious adverse event occurred during the study and involved one care partner being re-admitted to hospital due to a pre-existing condition. Change in the MAAS (Brown & Ryan, 2003) data suggests increased mindfulness for four stroke survivors (80%) and four care partners (80%), whilst one stroke survivor (20%) reported a reduction in mindfulness during the study. Interpersonal changes found in the MS data suggest improved mutuality for two stroke survivors (40%) and a care partner (20%) (Archbold et al., 1990). A stroke survivor (20%) and three care partners (60%) experienced a lowering of mutuality. HADS anxiety sub-scale (HADS-A) data showed improved anxiety symptoms for four stroke survivors (80%) and three care partners (60%) (Zigmond & Snaith, 1983). One stroke survivor (20%) and one care partner (20%) experienced increased anxiety symptoms. MCID occurred for three stroke survivors (60%) and two care partners (40%). HADS depression sub-scale (HADS-D) scores showed improvement for three stroke survivors (60%) and one care partner (20%) (Zigmond & Snaith, 1983). One stroke survivor (20%) did experience a worsening of depressive symptoms. MCID was achieved by week four and sustained to week eight for two stroke survivors (40%) (Table 3).

### Qualitative Findings

#### Dialectical Tension

Dialectical tension was the main overarching theme produced in the analysis and is evident within each of the four sub-themes. Dialectical tension helps articulate the contradictory and opposing information within the findings. The final IPA themes were articulated using dialectical tension because it captured a range of experiences, the complexity of the phenomena, and the nuance associated with care partnerships using online MBI after stroke. Dialectical tension reflects the continuum of experiences within the four sub-themes: curious without being curious; using whilst not using; together and apart; and remaining the same and changing.

#### Curious Without Being Curious

The dialectical tension of curious without being curious is complex. Stroke care partnerships appear to be curious about online MBI and are curious enough to enrol into a study to access online MBI. The curiosity appears to be stronger with stroke survivors compared to care partners, with some care partners not being curious about online MBI.

“I thought the course was very useful for myself, at this point in my life, for someone with a stroke. I knew I needed something to help me, erm, mental and emotionally as they call it and hence why I contacted you originally” (stroke survivor 1).

“I always thought it was never something I was interested in. It is just when I see it, I thought, I will do it because I really think that is what my dad needs. I did do it but I feel I didn't personally get anything out of it, but I did it because I wanted to be able to speak to my dad. I could discuss it that way, but it is not something I would continue to be honest” (care partner 3).

Stroke survivors were also curious about online MBI and some had a strong preference for online delivery.

“Online sounds yes, you could actually do it when you wanted to do it, without having to … travel anywhere … [or] meet people” (stroke survivor 5).

Although not everyone liked online MBI, and some participants expressed a preference for a face-to-face group delivery.

“It is probably not the kind of thing that I would pick up on a regular basis … what I didn't like was not being able to engage with the person” (care partner 5).

This theme suggestion some stroke care partnerships were curious about online MBI and that they thought it was appropriate for people affected by stroke.

#### Using Whilst Not Using

Using whilst not using captures the complexity surrounding engagement and explores reasons for non-engagement. The experience of using online MBI is captured in the following excerpts where care partnerships describe the ease of using the online MBI to manage anxiety symptoms.

“Erm, I have struggled a couple of times in the hospital last month and I have had to use mindfulness to try and help me out of that immediate moment. I find myself getting really anxious, erm, that tends to link with me thinking that my blood pressure is going up” (stroke survivor 1).

There is evidence participants were experiencing MBI in different ways, with some adapting MBI practices and others developing their own unique ways of practicing MBI with stroke.

“I was struggling to keep my arm in that position for the length of time watching the lady do it. So I probably had to break off or do it more on my right had side. So I sort of adapted, erm but yeah after I got into my own rhythm or technique” (stroke survivor 1).

The reasons given for not using MBI varied, but included feeling MBI was a chore, lack of interest, or not feeling it was for them.

“It hasn't had that much affect in my life, know? I have just dipped in and out of it for a bit of interest. I haven't been that focused on it” (stroke survivor 5).

“It was, it was, I didn't get very far because it sort of became a chore. Whereas doing other things I am absorbed in, for me, that was much more beneficial” (care partner 5).

Initial analysis appeared to suggest stroke survivors were using online MBI whilst their care partners were not using online MBI. However, further analysis revealed that ‘using and not using’ was more complex. Many of the stroke survivors were using online MBI, but in a very specific way. What appeared to be happening was that the stroke survivors were using the online MBI to develop coping skills for future. The approach to using MBI as a coping strategy or safety net was most prominent with stroke survivors and contrasts with the recommendation of daily practice.

“I picked, I probably picked a couple of the techniques to use and do that. I wouldn't say that I sit down daily and do it now, but I know it is there and I get to use it, I have used it, probably, I want to try and get some techniques to use either when I am walking or when you have that spell in the car when you are doing nothing else, you know” (stroke survivor 1).

“I enjoyed it, as I said I have not finished the course yet. But I like the fact it is there and I can go to it. There are certain bits of it that I have taken out. It's like if I feel I am getting stressed I kind of centring myself, follow my breathing, and watch, er, and then I just feel as if, phewww, stress [indicates with hands that stress disappears]” (care partner 2).

It appears stroke survivors are more eager to use online MBI but tend to use it as a coping strategy and not as recommended in most protocols.

#### Together and Apart

This theme highlights the strong interpersonal connection within care partnerships prior to the study and mirrors the quantitative findings. Being together and apart when using online MBI represents the experience of stroke care partnerships being in relationships with high mutuality and commencing the study together, whilst also experiencing some separation when using online MBI. The pattern of practicing apart was the norm and evident with all care partnerships. On one occasion, the care partnership was very separate and apart when learning mindfulness.

“We did the work, we always worked separately, and we did have some chats, what each other was doing and what we thought of it. So I think it is definitely beneficial to have somebody, just to kind of bounce off each other, how you felt about it” (care partner 3).

“We didn't do it together as such, because I didn't get that much out of it, I don't have any feelings about doing it alongside each other. We didn't really discuss it much did we?” (care partner 5).

The mutuality evident in the care partnerships appears to have contributed to them becoming involved in the study and motivated them to learn MBI together. However, the stroke care partnerships completed the course apart and not with their care partner. Superficial interpretation might suggest participants completed the course as individuals alongside each other, but it appears the interpersonal dimension was more nuanced.

“I think so because, you know [husband] didn't really do the course as much as I did, but he was there at my side. Erm, so I was able to tell him how things were going, what I had been doing …” (stroke survivor 4).

“Well it was good because I was able to ask my dad, have you done this exercise, oh I am away behind, so we were kind of comparing where we were and what we were doing and we would discuss the different exercises. What ones he liked. I couldn't get to the 30 min of the body scan, so we used to just talk about that, 30 min was too long for me. We kind of discussed things like that, but I definitely think it is something, it has been a positive thing to do, because we know when my dad did do it is being helpful, it was helpful” (care partner 3).

These excerpts demonstrate the stroke survivors, and their care partners did not complete the course simultaneously but highlight how they were working together. This shows the care partner's role was primarily supportive and encouraging for the stroke survivor. It also shows both the stroke survivor and care partners thought having a partner was helpful when using online MBI.

“You need somebody, because if I was doing it on your own and your partner thought it was a load of mumbo jumbo and you went into the corner and practice your mindfulness, yes it would be hard. Because really you need to be quite open to say, I’m just going to do this. So if you are getting that support, it makes the whole process much easier” (stroke survivor 1).

“I was still engaging with it because, I am consciously aware of [wife] all the time, erm and that's it. Something might not work for me, but if it works for her, then I pay attention” (care partner 4).

Another way care partners supported stroke survivors was by listening to their experiences and talking to them about MBI. This communication appears to have created an opportunity to reflect on the experience of using MBI.

“Erm, I kept my wife [name removed] in the loop of where I was, she was part of it, she was asking me about the mindfulness and what I was doing at the time” (stroke survivor 1).

The interpersonal dimensions are subtle and complex and appear to represent a dialectical tension between being together and being apart when learning online MBI.

#### Remaining the Same and Changing

This theme illuminates a dialectic experienced by participants who continue to experience anxiety and depression (remaining the same), whilst also reporting changes in the level of anxiety and depression (changing). The theme reveals the conflicting nature of some findings and highlights subtle changes in experiences of anxiety and depression.

“It is probably good in a way because of what has gone on since we last met, it has been more stressful, more anxiety, so that if I didn't have this mindfulness going on it might have been worse” (stroke survivor 1).

Continued anxiety and depression suggest participants are remaining the same. However, remaining the same is only one side of the dialectic and participants also reported experiencing changes in anxiety and depression when using online MBI.

“Yeah, mindfulness has helped with that thought process. Trying to break, if you are sort of having a bad spell, going into a mindfulness technique to try and break that train of thought, that has helped and I have had a couple of incidences when, it seems to have worked at that moment in time. My anxiety level just dropped totally dealt with the situation in the hospital and she got out later on that evening” (stroke survivor 1).

Several participants were able to describe how their level of mindfulness had changed since using the course. It is important to notice the changes in mindfulness reported by participants are articulated in terms of developing positive psychological wellbeing rather than the reduction of distress.

“For myself, I would say it is more kind of a serenity and an acceptance of your circumstances. I just get a peacefulness, you switch off to everything else around you and you just you know. I would not say for myself it is like concentrating on myself, but as I say you just kind of sit there and you just accept where you are, in that moment, erm everything around you just switches off” (care partner 4).

“I don't know really just, I do it just throughout the day type of thing, just paying attention to flowers in the garden, bumble bees in the lavender, yeah just paying attention to what is going on” (stroke survivor 4).

The experience of mindfulness for participants appears to be closely linked to increases in awareness and acceptance developed through using online MBI together. This reveals the complex and sometime contradictory experience of stroke care partnerships using online MBI.

## Discussion

This is the first study to investigate online MBI with care partnerships experiencing anxiety and depression following stroke (Parkinson et al., 2019; Parkinson, 2021). The study uses mixed methods case study research and combines an initial quasi-experimental stage followed by IPA (Creswell & Plano Clark, 2017; Smith et al., 2009). This design used explicit qualitative methods and is an example of a novel qualitative-orientated mixed methods design (Mayoh & Onwuegbuzie, 2015). Five stroke care partnerships (10 participants) used online MBI and experienced improved mindfulness and mutuality. A recent systematic review examined the efficacy of recruitment into stroke rehabilitation RCTs between 2005–2015 and found studies enrol one or two participants per site each month (McGill et al., 2020). The recruitment rate in this study was one participant per month, but this was achieved in the context of an unfunded PhD project recruiting stroke survivors and their care partners together.

Appropriateness and acceptability of online MBI for care partnerships affected by stroke was evaluated by calculating the number of module completed. There is limited research into factors that might affect stroke survivors’ engagement with online MBI, but it appears to be influenced by participant motivation, conscientiousness, and trait mindfulness (Forbes et al., 2018). Stroke care partnerships’ opinion towards online MBI varied and appears to represent a dialectic between being curious whilst not being curious. Some participants saw MBI as a possible solution for their mental health difficulties and something they could use as a coping strategy when highly stressed. The perception of online MBI being a useful coping strategy for psychological distress appears a common experience and is often reported in the literature (Schanche et al., 2020). However, not everyone thought it would be useful and some care partners did not think it was something they would use. Interestingly no participants thought MBI was inappropriate and/or unacceptable for stroke care partnerships, but the degree of appropriateness/acceptability varied within and between stroke care partnerships. Participants liked the facilitated nature of the MBI with several participants referring to the recorded therapist by name during the interviews. Participants also liked the fact it was asynchronous so they could engage at a time and pace of their choosing. These findings echo another survey which found the majority participants (81%) were willing to use online intervention, but most preferred guided interventions (39%) over non-guided interventions (19.2%) (Apolinário-Hagen et al., 2018).

The impact of using online MBI was most evident in the remaining the same and changing theme. The theme reflects an evolving dynamic process, which may change over a period of time as people become more (or less) mindful. A meta-ethnography (n = 14 studies) discovered participants learning MBI go through distinct phases: perceived safe certainty, safe uncertainty and grounded flexibility (Malpass et al., 2012). Results in this study suggest mutuality increased for some participants when using online MBI together, although, relationship quality may increase organically after stroke and without using MBIs (Simeone et al., 2016). It was interesting to observe none of the participants practiced formal (e.g., sitting meditation) together, but some would engage in informal (e.g., mindful walks) MBI practices together. It is unclear whether concurrent formal MBI practice (e.g., sitting meditation as pair) would have been beneficial, because it is mainly used with healthy couples seeking to enhance their relationship (Carson et al., 2004). Moreover, a study using concurrent formal MBI practice with care partnerships affected by cancer (Price-Blackshear et al., 2020) found that interpersonal relationships did not improve during the study.

The analysis revealed some participants continue to experience anxiety/depression symptoms, whilst also reporting changes in the nature and extent of anxiety/depression symptoms. The experience of getting better but wanting to improve further is a common experience for participants using MBI and was recently reported in a qualitative study exploring change in a clinical trial (Schanche et al., 2020). The remaining the same and changing theme highlights that participants’ difficulties remained the same whilst also showing some signs of improvement. The theme remaining the same and changing echoes with another dialectical tension. Change vs non-change dialectical tension exists when people want MBI to alleviate unwanted symptoms instead of developing a more accepting relationship with difficulties (Sauer et al., 2011). This distinction is at the very heart of MBI practice, which tries to achieve acceptance rather than amelioration of difficulties (Kabat-Zinn, 2013). It also has implications for how success is measured with online MBIs because symptoms reduction may not be the most important component and thinking holistically about change and wellness might prove more useful (White, 2014).

### Limitations

This is a small self-selecting group of participants, so caution is needed when interpreting the findings. Self-selecting participants can be more motivated to engage with an intervention and may not reflect people seen in routine practice (Parsons et al., 2020). Other limitations include the lack of formal cognitive assessment, therefore, it is difficult to know whether anyone in the study had cognitive impairment and how cognitive impairment may have affected engagement with online MBI. Another limitation was the lack of formal monitoring of home practice, which makes it difficult to know the amount and type of home practice undertaken by participants. The study also experienced a large amount of missing data. Missing data can adversely affect the validity of a study but was mitigated by using a combination of imputation and pairwise deletion methods. Possible confounders discovered during the post-intervention interview (week 8) include one stroke survivor starting anti-depressant medication and one care partner commencing additional non-mindfulness activities, which appear to have had a positive impact of their mood.

### Implications for Practice

Nurses providing holistic nursing care for stroke survivors and their care partners need to consider the possibility of post-stroke anxiety/depression. Stroke survivors and their care partners can feel abandoned by services and post-stroke anxiety/depression is frequently undertreated (Hackett & Pickles, 2014; Ayerbe, 2015; Dar et al., 2017; Ladwig et al., 2018). Online MBI could form a valuable part of holistic nursing practice for stroke survivors and their care partners, but more research is required before making recommendations for practice. Future research could compare online MBI with individual stroke survivors against online MBI with care partnerships. This direct comparison would help determine what value (if any) the partnership element provides for participants. Other recommendations for research include using home practice diaries or electronic recording devices to monitor the quantity and quality of home practice (Parsons et al., 2020). Stroke care partnerships may also need more time to complete online MBI and incentives could help improve adherence and/or encourage participants to complete data collection (Querstret et al., 2017).

The online MBI used in this study was not designed for people affected by stroke and it would be helpful to tailor online MBIs for people affected by stroke. The Helping Ease Anxiety and Depression after Stroke (HEADS: UP) research programme has adapted MBSR for people affected by stroke and has been delivered to stroke survivors and their care partners together using online interactive group sessions (Lawrence, 2019). Currently there is no stroke specific version of MBCT (Teasdale et al., 2000) and future research could involve developing online MBCT for people affected by stroke. Another option for future research would be to take the learning from this study and further adapt the HEADS: UP programme for asynchronous (i.e., pre-recorded) online delivery for people affected by stroke.

This study found poor engagement with online MBI by care partners, but value for stroke survivors using online MBI in a care partnership. Future research could explore ways of helping care partners engage with online MBI and/or investigate how care partners can support stroke survivors using online MBI without care partners having to complete the online MBI themselves. This might involve stressing the potential value of online MBI for care partners, developing orientation sessions and/or other resources (e.g., manuals) for care partners who want to support a stroke survivor using online MBI. This could help maximise support for stroke survivors and reduce the additional burden for care partners who do not want to attend online MBI. It is recommended stroke survivors and their care partners have access to MBI and different methods of delivery (e.g., online, in-person) because personal preference is important and the same methods of delivery may not suit everyone (Wahbeh et al., 2016). Online MBI is also well placed for a post COVID-19 world and could help provide remote intervention for people affected by stroke who may otherwise have limited access to psychological support services.

## Conclusion

Online MBI is a holistic self-management intervention and appears appropriate for stroke survivors and their care partners. Some participants improved their levels of mutuality, mindfulness, anxiety symptoms, and depression symptoms. Clinically important improvements were achieved for anxiety symptoms and depression symptoms in some people. Not all participants experienced improvements and variation existed in how beneficial online MBI was for participants. The variation experienced by participants using online MBI provides a new interpretation of the complex and sometimes contradictory nature of care partnerships using online MBI together. This is a new area of study and the findings need to be interpreted cautiously.

## Supplemental Material

sj-docx-1-jhn-10.1177_08980101221135723 - Supplemental material for Online Mindfulness with Care Partnerships Experiencing Anxiety and Depression Symptoms after Stroke: Mixed Methods Case Study Research Click here for additional data file. Supplemental material, sj-docx-1-jhn-10.1177_08980101221135723 for Online Mindfulness with Care Partnerships Experiencing Anxiety and Depression Symptoms after Stroke: Mixed Methods Case Study Research by Ben Parkinson, Maggie Lawrence, Evelyn McElhinney and Jo Booth in Journal of Holistic Nursing
