# Supplementary material for: Online Mindfulness with Care Partnerships Experiencing Anxiety and Depression Symptoms after Stroke: Mixed Methods Case Study Research
Source: J Holist Nurs. 2022 Nov 9;41(2):185–99. doi: 10.1177/08980101221135723 (PMC10236236; doi:10.1177/08980101221135723)
Supplement: sj-docx-1-jhn-10.1177_08980101221135723 - Supplemental material for Online Mindfulness with Care Partnerships Experiencing Anxiety and Depression Symptoms after Stroke: Mixed Methods Case Study Research [file sj-docx-1-jhn-10.1177_08980101221135723.docx]

**Automated Data from Be Mindful**

| **ID** | **PSS**  **Start** | **PSS**  **Post** | **PSS**  **F/U** | **GAD7**  **Start** | **GAD7**  **Post** | **GAD7**  **F/U** | **PHQ9**  **Start** | **PHQ9**  **Post** | **PHQ9**  **F/U** |
| --- | --- | --- | --- | --- | --- | --- | --- | --- | --- |
| SS1 | 20 | 20 | 19 | 5 | 6 | 6 | 6 | 6 | 3 |
| CP1 | 18 |  |  | 7 |  |  | 8 |  |  |
| SS2 | 23 | 12 | 13 | 11 | 0 | 1 | 11 | 0 | 2 |
| CP2 | 20 |  |  | 6 |  |  | 3 |  |  |
| SS3 | 21 | 21 |  | 6 | 10 |  | 8 | 16 |  |
| CP3 | 12 |  |  | 4 |  |  | 1 |  |  |
| SS4 | 5 | 6 | 5 | 2 | 2 | 2 | 5 | 3 | 2 |
| CP4 | 12 |  |  | 1 |  |  | 2 |  |  |
| SS5 | 9 |  |  | 6 |  |  | 3 |  |  |
| CP5 | 14 |  |  | 1 |  |  | 4 |  |  |
| Key  F/U = follow-up  SS 1-5 = stroke survivor  CP 1-5 = care partner  PSS = Perceived Stress Scale (Cohen et al., 1983)  GAD7 = Generalised Anxiety Disorder-7 (Spitzer et al., 2006)  PHQ9 = Patient Health Questionnaire-9 (Kroenke, Spitzer and Williams, 2001) | | | | | | | | | |
